# Close-Range Tracking of Underwater Vehicles Using Light Beacons

**DOI:** 10.3390/s16040429

**Published:** 2016-03-25

**Authors:** Josep Bosch, Nuno Gracias, Pere Ridao, Klemen Istenič, David Ribas

**Affiliations:** Computer Vision and Robotics Group, Centre d’Investigació en Robòtica Submarina, Parc Científic i Tecnològic, Universitat de Girona, 17003 Girona, Spain; ngracias@eia.udg.edu (N.G.); pere@eia.udg.edu (P.R.); klemen.istenic@udg.edu (K.I.); dribas@udg.edu (D.R.)

**Keywords:** tracking system, AUV, relative navigation, pose estimation, light beacons, active markers

## Abstract

This paper presents a new tracking system for autonomous underwater vehicles (AUVs) navigating in a close formation, based on computer vision and the use of active light markers. While acoustic localization can be very effective from medium to long distances, it is not so advantageous in short distances when the safety of the vehicles requires higher accuracy and update rates. The proposed system allows the estimation of the pose of a target vehicle at short ranges, with high accuracy and execution speed. To extend the field of view, an omnidirectional camera is used. This camera provides a full coverage of the lower hemisphere and enables the concurrent tracking of multiple vehicles in different positions. The system was evaluated in real sea conditions by tracking vehicles in mapping missions, where it demonstrated robust operation during extended periods of time.

## 1. Introduction

Oceanographic exploration and research are still today challenging tasks due to the demanding conditions underwater. The use of remotely-operated vehicles (ROV) and autonomous underwater vehicles (AUV), especially in deep-water operation, is essential for applications as varied as environmental surveying, geology, archeology, cable inspection and several others relating to industry and the military. However, the existing technology is still immature for close-range surveying of rugged terrain, such as caves, narrow passages or overhangs, due to limitations on the terrain sensing and on the navigation accuracy.

The use of a team of robots navigating in a close formation has the potential to significantly expand the coverage swath in mapping missions that require close proximity to the seafloor, such as optical or electromagnetic surveying. In areas of high topography, rigid arrays of sensors cannot be used safely, whereas AUV formations can provide the required degree of terrain compliance. The present work has been developed within the framework of the MORPH ( Marine robotic system of self-organizing, logically linked physical nodes) EU-FP7 project (2012–2015) described in [1]. This project proposes a novel concept of an underwater robotic system that emerges out of using different mobile robot modules with distinct and complementary resources. These mobile robots navigate at a very close range as a group and have the ability to adapt the formation to changes in the terrain. The most relevant concept with respect to this paper is that an underwater vehicle equipped with a multibeam sonar profiler advances at the forefront of the formation, flying at a “safe” altitude from the sea-floor, while two other vehicles fly behind, very close to the bottom, acquiring images. As can be deduced, precise knowledge of the poses of all robots during the missions is fundamental for both safe navigation and an accurate reconstruction of the optical and acoustic maps. The relative localization between vehicles is done through acoustic ranging. There has been space, though, for experimenting with the use of a vision-based method as an alternative for relative localization at short distances, where acoustics cannot provide updates with enough precision and frequency to ensure safety.

**Figure 1:**
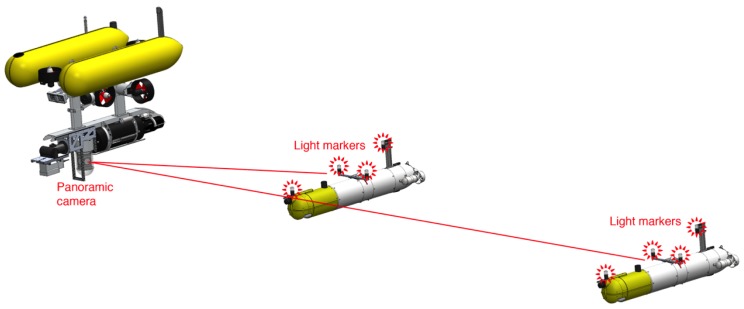
Representation of the localization of two target vehicles using active light markers. The leading vehicle, endowed with a wide field of view camera, localizes two target vehicles that are equipped with a set of active light markers.

Under adequate visibility conditions, optical cameras can be very effective for computing precise position estimates, including full inter-vehicle poses. The effects of absorption and scattering often preclude the use of standard feature detectors [2] as a solution to the problem of vision-based formation sensing. To improve the chances of detecting point features and to identify individual vehicles, this paper proposes to endow the AUVs with light beacons, namely a set of active light markers blinking with distinctive patterns to facilitate their recognition. With this system, it is possible to track vehicles with full information about their relative pose with high accuracy and rapid update rates. In order to have a sensor with the widest possible field of view, an omnidirectional underwater camera was used to provide full vision of the lower hemisphere during the experiments (Figure 1).

This paper presents all of the aspects related to the system: the components and methodologies used, as well as the experiments performed and the results obtained.

### 1.1. Related Work

Navigation and localization are two of the most important topics for underwater robotics. While navigation in land and air robotics is mainly based on the use of GPS and inertial sensors, the inability to receive GPS updates underwater makes the task of navigating precisely more challenging [3]. Most AUVs rely on the use of inertial sensors combined with a Doppler velocity log (DVL) [4], an acoustic-based instrument that measures relative velocities with respect to the water or ground. However, this navigation technique is subject to drift over time. To avoid the unbounded growth of the navigation error in long missions, the system must restart the navigation periodically either by surfacing and receiving a GPS update [5] or by determining its relative position from an external reference point. It is at this point that acoustics are highly relevant. With acoustic ranging, it is possible to determine the relative position between an AUV and one or multiple beacons placed in a known underwater position. Between all existing acoustic technologies, the most widely used are long baseline systems (LBL) and ultra-short baseline systems (USBL) [6,7].

An LBL system [8,9] comprises two or more geo-located beacons, which are usually attached to the seafloor. Whenever these beacons receive an acoustic signal from an AUV, they reply to it after a short known delay. With the knowledge of the two-way time-of-flight time of the signals, the position of the beacons and the speed of sound, it is possible to precisely localize an underwater vehicle. An USBL system [7,10], instead, consists of a single acoustic beacon, which is localized by an array of transceivers able to estimate both the range and angles of the incoming signal and, hence, the relative position of the beacon. This system can be used for tracking an AUV from a vessel (where the transceiver array is placed) or for improving the navigation system of the AUV, placing the array in the vehicle and the beacon in a geo-located position.

A solution to the underwater relative position measurement for multiple AUVs, was developed within the framework of the European GREX (Coordination and control of cooperating unmanned systems in uncertain environments) project (2006–2009) [11,12]. The navigation systems of each vehicle were combined with acoustic ranging from modems, to keep formation while following a predefined path. This achievement paved the way for underwater applications featuring multiple AUVs. Formation flying was limited to areas with approximately flat seafloors due to constraints on the vehicles pre-planned formation and to ensure reliable use of DVLs. Over rough terrain, DVLs tend to be highly inaccurate, and are of limited use for ensuring vehicle safety in tight formations. In the TRIDENT (Marine Robots and Dexterous Manipulation for Enabling Autonomous Underwater Multipurpose Intervention Missions) project (2010–2013) [13], a homing and docking solution using a USBL was tested. An intervention AUV (I-AUV) [14] was placed in charge of a survey and intervention mission, while an autonomous surface craft (ASC) was employed at the surface for communications purposes. Once the intervention mission was finished, the I-AUV (fitted with the USBL transceiver array) started a homing and docking procedure in order to dock in a special structure in the ASC, where the USBL beacon was located.

The use of easily-identifiable light sources for pose estimation has gained momentum in recent years in applications of land and aerial robotics. Recent examples are the work of Censi *et al.* [15] and Faessler *et al.* [16], where favorable visibility conditions allow the use of fast cameras and infrared LEDs to provide very fast pose updates. However, in underwater applications, where the detection and identification of the light sources is far more challenging, few attempts have been made.

Krupinski *et al.* presented [17] a docking panel equipped with active light markers as an alternative to acoustic localization for close ranges. Li *et al.* [18] applied this concept in a docking station for underwater vehicles. Four green LEDs were placed along a large funnel to make it visible to underwater vehicles using a stereo camera. Nevertheless, as there was no necessity of estimating the orientation nor the motion of the docking station, all of the green LEDs were permanently lit, and no identification was necessary. The fact that in our case the markers are placed on a mobile target makes it essential to distinguish and identify each one of the beacons to predict the motion accurately.

### 1.2. Contributions

The main contributions of this paper are:

1. A new method for pose sensing and relative navigation for multiple AUVs in short ranges based on the use of active light markers. This method has the following advantages in comparison with traditional systems:
  (a) **High rate pose estimation**: The update rate depends on the frame rate of the camera and the capacity of the computer in charge of processing the images. It is expected then that the update rate can be higher in the near future with the rapid evolution of computer technologies.
  (b) **High precision**: The minimum number of markers to retrieve the pose of a target vehicle is three. When using only three markers, the accuracy of the estimated pose depends strongly on the position of the markers on the vehicle and its location relative to the observer. The use of extra markers drastically reduces the uncertainty of the poses obtained, making the technique suitable for applications where very good accuracy is needed, such as cooperative underwater manipulation. A second source of uncertainty has to do with the location of the beacons in the image. The rapid evolution of underwater cameras in terms of resolution and sensitivity will lead to further improvements in the pose accuracy.
  (c) **Relative orientation data**: The most frequently-used acoustic localization systems, such as USBL or LBL, provide information about the relative position of a target, but they cannot provide information about its orientation. The light beacon system is able to provide this information with little uncertainty.
  (d) **Low-cost**: Another handicap of acoustic-based systems is their cost. Acoustic modems and arrays are significantly more expensive when compared to optical cameras. The decreasing price of cameras makes the approach described in this paper extremely competitive against traditional systems.
2. Real experiments and results of the method presented: The system was tested at sea over several missions with results that support the advantages listed above. In the experiments presented in this paper, the filtered pose estimates were updated at approximately 16 Hz, with a standard deviation lower than 0.2 m in the distance uncertainty between vehicles, at distances between 6 and 12 m.

The rest of the paper is organized as follows. Section 2 describes the different components of the system. Section 3 presents the approach followed for the tracking process. In Section 4, the results of the experiments for testing the capacities and reliability of the whole system are presented. In the last Section 5, we present some conclusions.

## 2. System Description

The objective of the method is the real-time localization of underwater vehicles for distances less than 10 m and to obtain both position and orientation information with high update rates. The resulting system must also be robust to short temporal occlusions of the direct line of vision to the target markers.

The proposed solution consists of the placement of a set of light beacons, or active markers, on the target vehicles, which are optically tracked by a wide field of view camera placed in a camera vehicle. The tracking of these markers allows estimating the 3D pose of the target vehicles. Tracking of multiple target vehicles is possible by using different blinking pattern frequencies. The underlying assumptions are that the camera field of view covers the areas where the vehicles operate and that the visibility conditions are not severe for the intended inter-vehicle distances.

The light beacons and the camera system are the two main hardware components and are detailed in the following section.

### 2.1. Light Beacons

**Figure 2:**
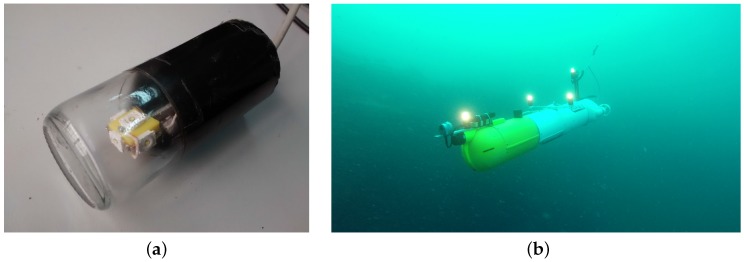
Detail of a light beacon and demonstration of a light beacon set assembly in a real AUV. They can be easily installed and adapted to any vehicle geometry. (**a**) Details of an active marker. Each one consists of five high-intensity LEDs; (**b**) The Sparus II AUV [20] equipped with a set of four active markers during a mission.

Each set of beacons consists of four markers connected through electric cables to a control board inside a watertight housing. This housing is placed in the payload area of the vehicles and is powered by the batteries of the robot. This setup makes it easy to install the markers on different vehicles and to distribute them in different geometries according to the vehicle design (Figure 2b). It is essential to have a precise measurement of the location of the markers with respect to the navigation origin of the vehicle for correct operation of the system. Each individual light beacon consists of five high-intensity LEDs oriented strategically to create a homogeneous omnidirectional lighting effect inside a cylindrical waterproof housing (Figure 2a). The system is operated at 24 V, and the maximum power consumption is 22 W, when all markers are lit.

The number of beacons used and how they are placed on a target vehicle are two factors that directly influence the precision of the estimated pose. A comprehensive study of such factors can be found in [19].

**Figure 3:**
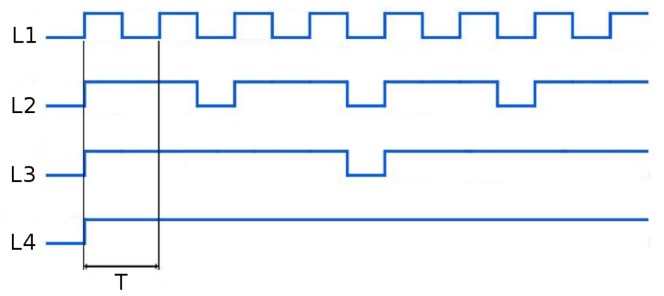
Blinking pattern of each marker of the light beacon set. Each beacon has a distinctive pattern allowing the global identification in the minimum time possible while there are at least three beacons lit more than 50% of the time.

Various possibilities of differentiating each individual marker of the set to allow its identification were studied. One of the most popular and simple techniques used in land robotics is the use of colors to distinguish the different beacons composing the set, but this option was discarded, due to the difficulty in consistently discerning colors for light sources at distances larger than a few meters. The use of different blinking frequencies for each individual marker was discarded due to the use of this strategy for identifying different sets of light markers, and hence, being able to estimate the pose of different vehicles simultaneously. Instead, different blinking patterns, illustrated in Figure 3, are used to allow the identification of the different markers in each set.

Two objectives were considered in designing the patterns. On the one hand, it should allow the identification of all lights in the minimum time possible, and on the other, it should maximize the time when all lights are on, thus allowing the pose estimation algorithm to compute a large number of poses per second, which facilitates tracking.

Two different sets of active markers with different minimum cycle periods, *T*, were manufactured to allow the simultaneous tracking of two target vehicles on the same mission. These periods must be chosen according to the camera frame rate, *fps*, and the design requirements, as they implicitly define the minimum time necessary for the identification of the light beacons. The minimum cycle period, *T*, contains for the fastest marker (L1) one *on* and one *off* period, and we need to be sure that the camera captures at least one frame and preferably two to improve robustness: one where the beacon is lit and another where the beacon is off. Thus, $\frac{T}{2}>\frac{1}{fps}$.

For the experimental setup used in this paper, the camera has a frame rate of 16 fps, and so, T>0.125 s. The values used for the first and second sets of light beacons were $T_{1}=0.25$ s, and $T_{2}\;=\;0.7$ s, respectively.

The minimum time needed for the identification of *n* markers, $T_{ident}\left(n\right)$, is proportional to the minimum cycle period. For the pattern presented in Figure 3, this time is $T_{ident}\left(n\right)=2^{n-1}\;T$. Thus, the minimum time necessary for the identification of three of the markers, $T_{ident}\left(3\right)$, which is the minimum necessary to compute a first pose estimation, is: $T_{ident}\left(3\right)=4\;T$.

### 2.2. Panoramic Camera

The camera used for the localization must have a wide field of view (FOV) in order to keep track of the target vehicle in a wide range of positions and orientations of both the target and the leading vehicle. For this reason, in the experiments presented in this paper, a panoramic (or omnidirectional) camera was used instead of a conventional one.

#### 2.2.1. Model and Assembly

**Figure 4:**
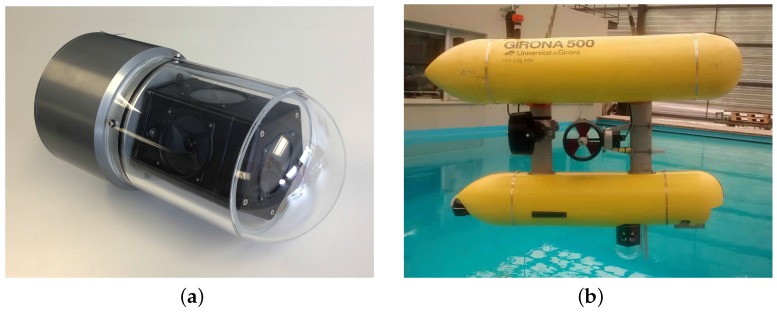
Omnidirectional camera and assembly in Girona500 AUV. The camera was installed under the bottom cylinder to have a clear view of the bottom hemisphere and is protected by two aluminum bars installed in front of the camera. (**a**) Omnidirectional underwater camera based on a Point Grey’s Ladybug camera and a custom waterproof housing; (**b**) the Girona500 AUV equipped with the omnidirectional camera.

The camera used is an omnidirectional multi-camera system (OMS), based on a Point Grey’s Ladybug 3 [21]. The Ladybug 3 comprises six individual cameras and is designed for land-based applications. A custom housing was designed to make it submersible up to 60 m (Figure 4a). The housing is composed of a transparent poly-methyl methacrylate (PMMA) dome, which contains the camera, and an aluminum alloy body, which contains a small form factor computer dedicated to processing the video feed. The computer is connected directly to the Ladybug 3 through a FireWire 800 Mbps connection. The housing has a single external Ethernet cable used for both power and communications.

For the experiments presented in Section 4, the omnidirectional camera was mounted in the bottom part of Girona500 AUV [22], as shown in Figure 4b. To protect the camera from any damage in the unlikely event of a collision, two aluminum bars were placed in front of the camera. These bars have no impact on the performance of the tracking system, as the target vehicles were always behind Girona500 in the formation employed during the missions. The Girona500 is able to power up or down the camera through a digital output and communicates through Ethernet with the computer embedded inside the housing, which provides the estimated poses of the target vehicles.

#### 2.2.2. Camera Calibration

The camera outputs six separate images that can later be combined to create a hemispherical panorama or treated separately as individual images according to the mission objectives. In both cases, the camera must first be calibrated to ensure proper use of the images collected. The calibration takes into account all of the distortions introduced by both the lenses of the camera and the waterproof housing, as well as the relative positioning between the individual cameras. The calibration of such a complex camera was divided into three different steps: intrinsic, extrinsic and underwater calibration.

The intrinsic parameters of all single cameras are necessary to project a 3D point in space onto the 2D image plane. They depend on specific geometry properties of each camera, as well as lens properties, such as focal length ($f_{l}$), principal point (*c*) or distortion coefficients. The pinhole camera is the most used camera model due to its compactness and freedom from distortions [23]. However, all lenses introduce image distortions that are not explicitly included by this model. The most common one is radial distortion, which is due mainly to the shape of the lenses and produces nonlinear distortions along the radial direction from the principal point. The calibration of the intrinsic parameters is done separately for each single camera in air and without the waterproof housing, making use of a standard calibration toolbox. It is important to note that due to the high distortion of the lenses used in the Ladybug3 camera, a fisheye distortion model was used to properly correct the radial distortion.

The calibration of the extrinsic parameters consists of the determination of the exact geometric relationship between the different camera frames composing the OMS. For this calibration, a specific procedure was developed. This procedure was based on a bundle adjustment of multiple features observed from different images, similar to the calibration of a stereo camera. The data necessary for this calibration were collected in air and without the waterproof housing.

**Figure 5:**
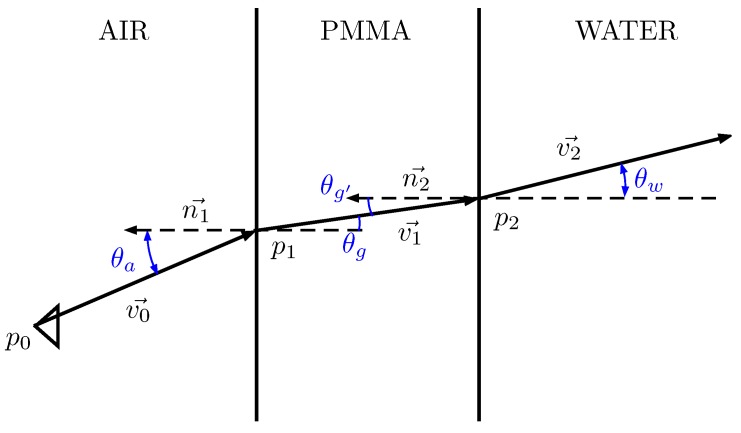
Ray tracing schematic of a single optical ray passing through air, PMMA and water.

The underwater calibration consisted of determining the exact position and orientation of the waterproof housing with respect to the camera. It is worth noticing that the direction of the rays changes at every medium transition found along the path from the imaging sensor inside the camera to a point underwater (Figure 5). A small error in the relative position of the housing can lead to a big inaccuracy in the direction of the final ray.

Once the multi-camera system has been calibrated, it is possible to obtain the projection function *f*, which projects a 3D point into a 2D location in the image sensor of a chosen camera, and its inverse $f^{-1}$, which projects a 2D pixel of an image sensor onto a 3D ray.

For a conventional pinhole camera, the function $f^{-1}$ for projecting a 2D pixel, $u=[u_{x},u_{y}]$, from a non-distorted image onto a 3D ray with center at the origin of the camera and direction vector $v=[v_{x},v_{y},v_{z}]$, is straightforward:

(1) $$v=\left[\begin{array}{ccc} \frac{u_{x}-c_{x}}{f_{l}}, & \frac{u_{y}-c_{y}}{f_{l}}, & 1 \end{array}\right]\;$$

where $c=[c_{x},c_{y}]$ is the location of the principal point in the imaging sensor and $f_{l}$ is the focal length.

For underwater cameras, the fact that the direction of the ray changes in every medium transition makes the process more laborious, as for each transition, the intersection point and the direction of the rays must be computed according to the laws of physics. For the sake of simplicity, the details are not described here, but can be found in [24].

For the case of the projection function *f* that projects a 3D point $p=[p_{x},p_{y},p_{z}]$ into a 2D location in the image sensor, it is equally simply to find an expression for pinhole cameras:

(2) $$u=\left[\begin{array}{cc} f_{l}\frac{p_{x}}{p_{z}}+c_{x}, & f_{l}\frac{p_{y}}{p_{z}}+c_{y} \end{array}\right]$$

In contrast, it is not possible to find such an expression for projecting an underwater 3D point. To solve this problem, an iterative process is run instead. This process goes along the pixels of the sensor and selects the one whose associated 3D ray passes closer to the desired 3D point.

## 3. Approach

**Figure 6:**
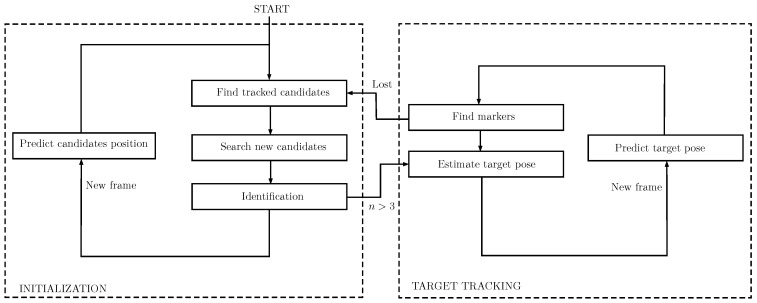
Block diagram of the light beacon tracking system.

The tracking of the target vehicle is divided into two stages (Figure 6). The first stage consists of an initialization step, where the pose of the vehicle is unknown and there is not enough information available for its estimation. The second stage begins when there is enough information for estimating the pose of the target vehicle, and it lasts until the tracking of the vehicle is lost, where the system returns to the initial stage.

During the initialization stage, the tracking consists of an independent tracking process for each of the lights. In contrast, the second stage, named *target tracking*, consists of the global tracking of the target vehicle.

All software programs have been implemented in C++ to achieve the best temporal performance possible and to make the tracking system able to work in real time. Different programming libraries have been used for the implementation of the full system, with special relevance on OpenCV [25] for the treatment of digital images and Ceres-Solver [26] for solving the non-linear least squares problems.

### 3.1. Initialization

During the initialization stage, there are three main tasks: (1) searching for new light candidates; (2) tracking previous candidates; and (3) deciding if they correspond to one of the beacons. When at least three candidate lights have been identified, the system moves to the second stage. During this stage, we will make use of acoustic ranges as extra information for making the prediction of the lights in future frames more precise, but this could be replaced by the assumed distance between the camera and the target vehicle for each mission.

#### 3.1.1. New Candidates

The system starts the identification process over the bright spots of the image. With this purpose, the gradient image is computed from the grayscale image using the Sobel operator. The Sobel operator applies two 3 × 3 convolution kernels to obtain two images that contain an approximation to the horizontal, $G_{x}$, and vertical, $G_{y}$, derivative images:

(3) $$G_{x}=\left[\begin{array}{ccc} -1 & 0 & +1\\ -2 & 0 & +2\\ -1 & 0 & +1 \end{array}\right]*A\;$$

(4) $$G_{y}=\left[\begin{array}{ccc} -1 & -2 & -1\\ 0 & 0 & 0\\ +1 & +2 & +1 \end{array}\right]*A\;$$

where * denotes the 2D convolution operation and A is the grayscale image.

The two derivative images are combined in order to obtain a gradient image:

(5) $$\left|G\right|=\sqrt{G_{x}^{2}+G_{y}^{2}}\;$$

**Figure 7:**
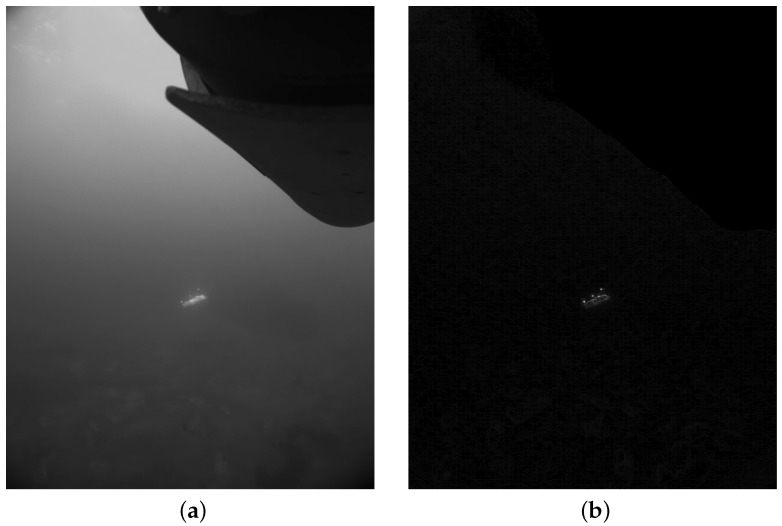
Sample image and its corresponding gradient image. (**a**) Original grayscale image; (**b**) gradient image with a mask for the part corresponding to the body of the AUV carrying the camera.

A mask can be applied to the resulting image with the aim of avoiding the further inspection of bright spots directly related to the body of the vehicle carrying the camera (Figure 7).

The next step consists of selecting from the gradient image the *n* brightest spots with a minimum distance *d* between them to analyse them in detail. For each one, we select a window in the original grayscale image, with size *w* centered on the point found previously, and we search for the local maximum closest to the center of the window, as the location may vary from the gradient image. Once the window has been re-centered and we are sure the candidate spot is in the center, we will check the following different conditions before accepting it definitely as a candidate light.

- **Intensity**: A minimum intensity value is required to accept a bright spot as a candidate light. This minimum depends on the existence of candidates in the previously-processed images. In the case that no previous candidates exist, the value depends both on the last acoustic range received and on an extra parameter reflecting the visibility (and sun conditions in case the mission is performed in shallow waters). In cases where there were candidates present in previous frames, the value of intensity required is slightly smaller than the minimum intensity of the existing candidates.
- **Size and shape**: For accepting the pre-candidate, its shape must be similar to a 2D-Gaussian distribution, and its area cannot be greater or lower than certain selected values. The first step required is determining the size of the spot analyzed. For this purpose, a technique very similar to the radial contrast function (RcF) method is used [27]. This algorithm was developed for source detection in astronomical images, but is flexible enough to be applicable to the images processed by our system. It operates by choosing the brightest pixel and analyzing the mean of the neighbor pixels at incrementally larger radial distances. The size of the light is determined when the intensity profile obtained stops decreasing and remains constant. Once the size is determined, we must ensure that its value is reasonable. As in the previous case, the minimum and maximum size values depend on the existence of candidates in the previous frames. In the positive case, the minimum and maximum values are determined from the features of the existing candidates, while in the other case, they depend on the last distance estimate available and the visibility conditions.

#### 3.1.2. Tracking of Candidates

Every time the system analyzes a new image, we look for lights that could correspond to previous candidates. To estimate the position of a previous candidate in a new image, we consider that the lights are still in the 3D space, and we take into account only the movement of the camera. It is important to note that any small rotation of the camera results in a significant displacement in the image; thus, assuming that the candidate lights are still in the image plane would result in a less efficient tracking.

The distance between the camera and the marker must be assumed in order to project the 2D marker position from the last image frame, uk-1=k-1[uxuy], to a 3D point, pk-1. In our case, that distance was assumed to be the last acoustic range, *r*, between the vehicle carrying the camera and the target vehicle,

(6) pk-1=f-1cam,r,k-1u

where *f* is the projection equation according to the camera calibration, as detailed in Section 2.2.2 and cam is the number of cameras.

Once the 3D point corresponding to the last frame, k-1, has been computed, it can be rotated and translated according to the transformation matrix, Tk-1k, which transforms a point from the k-1 coordinate system to the *k* coordinate system and is computed according to the navigation system of the camera vehicle:

(7) pk=kTk-1·k-1p

Finally, the rotated 3D point can be projected back to the image plane through the iterative method described in Section 2.2.2 to obtain the predicted position of the marker in the new image, uk:

(8) uk=fcam,r,kp

In the case that the predicted candidate position is close to the limit of the image, an image from one of the adjacent cameras will be selected accordingly before any further processing.

For each candidate light, we select a patch around its predicted position. The size of the patch, *w*, depends on the previous light size and the assumed distance *r*. In the patch, we search for the closest local maximum, and we again use the RcF technique described in the previous subsection. We check if there exist similarities between the candidate light and the local maximum found. Particularly, they must have similar intensity, size and intensity gradient, or otherwise, it is assumed that the maximum found does not correspond to the tracked light. Depending on the results obtained, we tag the candidate light as *on* or *off*. In the case that a candidate light has been tagged as *off* for a number of frames that is significantly larger than the expected number according to the beacons’ pattern, we remove it from the candidates list.

#### 3.1.3. Beacon Identification

After the tracking of the candidate lights, an identification method checks if any of the candidates can be associated with the beacons. The method described in this section has been proven very effective for the pattern described in Section 2, but may need modifications in the case of a different blinking pattern.

Each one of the tracked lights has a record of its full *on-off* state history. Especially important are the mean of the periods when the light was *off* (not detected) and the mean of the periods when the light was *on* (detected). A score matrix is computed to evaluate every possible association, containing as many rows as candidates and as many columns as markers. The matrix is initialized with a negative value for all cells, and two conditions must be met for computing a score value.

- An *off period* must have been detected for the candidate light, that is a light cannot be associated with a marker, if it has not been absent for at least one frame and detected again. Furthermore, the duration of this *off period* must be very close to the expected cycle time within a tolerance *t*.
- Additionally, for comparing a candidate light *i* with a marker *j*, the light must have been tracked for at least the duration of the marker full period; otherwise, we could not be sure the association is correct:
  (9) lighttrackingduration(i)>markerperiod(j)

If these two conditions are met, a score is computed for every possible association using:

(10) $$score\left(i,j\right)=1-\frac{expected\;time\;on(j)-mean\;time\;on(i)}{expected\;time\;on(j)}$$

Once all of the cell values are computed, we find the maximum value of the matrix. In the case that this value is greater than a certain threshold, 0.8 for the results presented in this paper, we identify the association as valid. The column and the row where the maximum occurs are removed, and a maximum is searched for again. If this maximum is greater than the threshold, the association is identified, and the corresponding row and column are removed. This process is repeated until all values of the matrix are lower than the threshold.

In the case that we have identified at least three lights, it is then possible to estimate the pose of the target vehicle. If the system was in the first stage of processing, it moves to the second one.

### 3.2. Target Tracking

Once a first pose estimation of the target vehicle has been computed, the system starts a tracking process over the target vehicle. The procedure is as follows:

1. Each time there is a new image acquisition, the elapsed time between the previous processed image and the new one is computed. A prediction of the movement of the target vehicle *S* with respect to the camera in the elapsed time is computed, taking into account both the motion of the camera and the dynamics of the target vehicle.
2. According to the predicted 3D pose of the target vehicle and the known position of the active markers along the vehicle’s body, each one of the markers is projected onto the image plane to obtain its 2D predicted position through the use of the projection equation (Equation (8)).
3. Each one of the markers is searched in the images according to its predicted 2D location using an identical process to the one described in Section 3.1.2. If at least three markers are detected, a new pose estimation is computed. Otherwise, the predicted pose is assumed to be the real one.

In order to reduce the noise in the estimated poses and obtain the smoothest possible dynamic model of the target vehicle, we make use of an extended Kalman filter (EKF). It has been found possible to reduce the noise for each one of the estimates and, thus, to produce a better result.

Details of the implementation of the EKF and the pose estimation are presented in the following subsections.

#### 3.2.1. Temporal Filtering

The use of an EKF filter proved very useful to reduce the noise of the estimated 3D poses of the target vehicle. At the same time, it allowed a better prediction of the 2D position of the markers in the images and significantly improves the performance of the system.

**State vector:**

**Figure 8:**
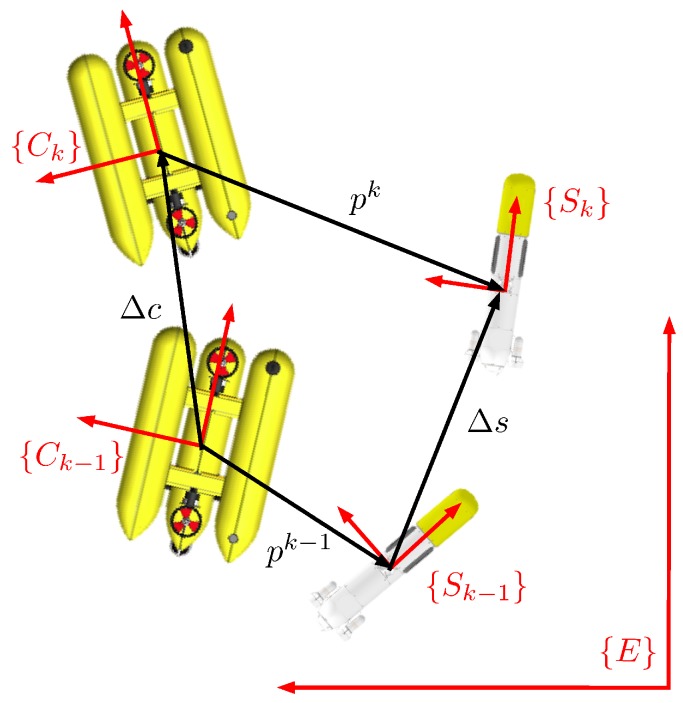
Representation of the target vehicle (*S*) and the camera vehicle (*C*) at instants *k-1* and *k*.

The EKF state vector has two different parts, $x_{k}={\left[\begin{array}{cc} p & \nu_{s} \end{array}\right]}^{T}$. The term *p* contains the six degrees of freedom defining the current position, $p_{1}$, and orientation, $p_{2}$, of the target vehicle *S* represented in the camera frame *C* at time *k* (see Figure 8):

(11) $$p={\left[\begin{array}{cc} {p_{1}}^{T} & {p_{2}}^{T} \end{array}\right]}^{T}={\left[\begin{array}{cccccc} x & y & z & \phi & \theta & \psi \end{array}\right]}^{T}\;$$

The term $\nu_{s}$ contains the six degrees of freedom defining the linear, $\nu_{1,s}$, and angular, $\nu_{2,s}$, velocities of the target vehicle with respect to the inertial frame *E* represented in the tracked vehicle frame $S_{k}$ at time *k*:

(12) $$\nu_{s}={\left[\begin{array}{cc} {\nu_{1,s}}^{T} & {\nu_{2,s}}^{T} \end{array}\right]}^{T}\;$$

**Prediction:**

Our model is governed by a non-linear function *f*:

(13) $$x_{k}=f\left(x_{k-1},u_{k},n_{k}\right)\;$$

which relates the state at a time *k*, $x_{k}$, given the state at a time k-1, $x_{k-1}$, a control input $u_{k}$ and a non-additive noise $n_{k}={\left[\begin{array}{cc} n_{1}^{T} & n_{2}^{T} \end{array}\right]}^{T}$ that follows a Gaussian distribution with zero mean and covariance $Q_{k}$.

According to the notation used in Figure 8, and assuming that the target vehicle follows a constant velocity model, *f* can be expressed as:

(14) $$x_{k}=\left[\begin{array}{c} p^{k}\\ \nu_{s}^{k} \end{array}\right]=\left[\begin{array}{c} \left(⊖\Delta c\right)\overset{A}{⊕}\left(p^{k-1}\overset{B}{⊕}\Delta s\right)\\ \nu_{s}^{k-1}+n_{2}\Delta t \end{array}\right]\;$$

where operators ⊕ and ⊖ denote the commonly-used six degrees of freedom inversion and compounding operations [28], the term Δt denotes the time elapsed between time k-1 and *k*, the term Δc denotes the variation of the pose of the camera vehicle in the elapsed time Δt and is part of the control input $u_{k}$, the term Δs corresponds to the variation of the pose of the target vehicle in the camera frame, $C_{k}$, and can be computed as:

(15) $$\Delta s=\left[\begin{array}{c} \nu_{1,s}^{k-1}\Delta t+\frac{1}{2}n_{1}{\Delta t}^{2}\\ J_{\omega }\left(p_{2}^{k-1}\right)\left(\nu_{2,s}^{k-1}+n_{2}\Delta t-R^{T}\left(p_{2}^{k-1}\right)\nu_{2,g}^{k-1}\right)\Delta t \end{array}\right]\;$$

where $\nu_{2,g}^{G_{k-1}}$ is the angular velocity of the camera vehicle at the instant k-1 and is part of the control input $u_{k}$, $J_{\omega }\left(p_{2}^{k-1}\right)$ is the Jacobian that transforms the angular velocity of the target vehicle (*S*) with respect to camera vehicle (*C*) to ${\dot{p}}_{2}^{k}={\left[\begin{array}{ccc} \dot{\phi } & \dot{\theta } & \dot{\psi } \end{array}\right]}^{T}$ and is given by:

(16) $$J_{\omega }\left(\phi ,\theta ,\psi \right)=\left[\begin{array}{ccc} 1 & \sin\phi \tan\theta & \cos\phi \tan\theta \\ 0 & \cos\phi & -\sin\phi \\ 0 & \frac{\sin\phi }{\cos\theta } & \frac{\cos\phi }{\cos\theta } \end{array}\right]\;$$

and $R^{T}\left(p_{2}^{k-1}\right)$ is the rotation matrix that transforms a point expressed in the *S* coordinate system to the *G* coordinate system which depends on their relative attitude $p_{2}^{k-1}$.

The prediction of the state ${\hat{x}}_{k}^{-}$ and its associated covariance $P_{k}^{-}$ are given by:

(17) $$\begin{array}{c} {\hat{x}}_{k}^{-}=f\left({\hat{x}}_{k-1},u_{k},n_{k}=0\right)\;\\ P_{k}^{-}=A_{k}P_{k-1}A_{k}^{T}+W_{k}Q_{k}W_{k}^{T}\; \end{array}$$

where:

(18) $$A_{k}=\frac{\partial f(x_{k-1},u_{k},n_{k})}{\partial x_{k-1}}{|}_{\left({\hat{x}}_{k-1},u_{k},n_{k}=0\right)}\;$$

and

(19) $$W_{k}=\frac{\partial f(x_{k-1},u_{k},n_{k})}{\partial w_{k}}{|}_{\left({\hat{x}}_{k-1},u_{k},n_{k}=0\right)}\;$$

According to Equations (14) and (15), the Jacobians $A_{k}$, $W_{k}$ are:

(20) $$A_{k}=\left[\begin{array}{cc} J_{2⊕}^{A}\left(J_{1⊕}^{B}+J_{2⊕}^{B}\left[\begin{array}{cc} 0_{3} & 0_{3}\\ 0_{3} & J^{*} \end{array}\right]\right) & J_{2⊕}^{A}J_{2⊕}^{B}\left[\begin{array}{cc} I_{3}\Delta t & 0_{3}\\ 0_{3} & J_{\omega }\Delta t \end{array}\right]\\ 0_{6} & I_{6} \end{array}\right]\;$$

(21) $$W_{k}=\left[\begin{array}{c} J_{2⊕}^{A}J_{2⊕}^{B}\left[\begin{array}{cc} \frac{1}{2}\Delta t^{2}I_{3} & 0_{3}\\ 0_{3} & J_{\omega }\left(p_{2}^{k-1}\right)\Delta t^{2} \end{array}\right]\\ I_{6}\;\cdot \;\Delta t \end{array}\right]\;$$

where $J^{*}$ can be obtained by symbolic differentiation of the following expression:

(22) $$J^{*}=\frac{\partial \left(J_{\omega }\left(p_{2}^{k-1}\right)\left(\nu_{2,s}^{k-1}-R^{T}\left(p_{2}^{k-1}\right)\nu_{2,g}^{k-1}\right)\Delta t\right)}{\partial (p_{2}^{k-1})}\;$$

**Measurement model:**

The general model for measurements is:

(23) $$z_{k}=H_{k}x_{k}+m_{k}\;$$

where $z_{k}$ is the measurement vector and corresponds to the relative pose of the target vehicle with respect to the camera:

(24) $$z_{k}={\left[\begin{array}{cccccc} x_{s}^{c} & y_{s}^{c} & z_{s}^{c} & \phi_{s}^{c} & \theta_{s}^{c} & \psi_{s}^{c} \end{array}\right]}^{T}\;$$

$H_{k}$ is the observation matrix and has the form:

(25) $$H_{k}=\left[\begin{array}{cc} I_{6} & 0_{6} \end{array}\right]\;$$

the term $m_{k}$ is a vector of white Gaussian noise with zero mean and covariance $R_{k}=\Sigma_{z_{k}}$. This covariance is provided by the pose estimation module (Section 3.2.2).

**Update:**

Updates happen when a new measurement is computed from the pose estimation module. Although the tracking system has been proven to be reliable, there are still situations where a misidentification or mismatching could cause the wrong calculation of the pose estimation and, thus, negatively affect the pose estimate of the filter. To avoid taking into account outlying observations, we check if the observation is consistent with the current prediction computing the innovation term, $r_{k}$, and its covariance, $S_{k}$:

(26) $$r_{k}=z_{k}-H_{k}{\hat{x}}_{k}^{-}\;$$

(27) $$S_{k}=H_{k}P_{k}^{-}H_{k}^{T}+R_{k}\;$$

Then, in order to determine the compatibility of the measurement, an individual compatibility (IC) test is performed. With this aim, the Mahalanobis distance [29] is computed as:

(28) $$D_{k}^{2}=r_{k}^{T}S_{k}^{-1}r_{k}\;$$

The observation is considered acceptable if $D_{k}^{2}<\chi_{d,\alpha }^{2}$, where the threshold $\chi_{d,\alpha }^{2}$ is given by the chi-squared distribution with *d* degrees of freedom and a cumulative value of 1-α. For the implementation in this paper, values of d=6 and α=0.05 were used.

Since the measurement model is linear, the classical KF equations can be used for the update; where the Kalman gain, $K_{k}$, is computed as:

(29) $$K_{k}=P_{k}^{-}H_{k}^{T}S_{k}^{-1}\;$$

and the estimate of the current state ${\hat{x}}_{k}$ and its covariance $P_{k}$ according to the Joseph form are:

(30) $${\hat{x}}_{k}={\hat{x}}_{k}^{-}+K_{k}r_{k}\;$$

(31) $$P_{k}=\left(I-K_{k}H_{k}\right)P_{k}^{-}{\left(I-K_{k}H_{k}\right)}^{T}+K_{k}R_{k}K_{k}^{T}\;$$

#### 3.2.2. Pose Estimation

The pose $x^{*}$ that best fits the observation of the markers in the image, *u*, is found using non-linear least squares minimization, by searching for the values of the variable *x* that minimize the sum of f(x):

(32) $$\min_{x}\frac{1}{2}\sum_{i}{∥f_{i}\left(x_{i_{1}},\cdots ,x_{i_{k}}\right)∥}^{2}\;$$

The variable *x* contains the complete pose of the target vehicle with respect to the camera $p={\left[\begin{array}{cccccc} x_{s}^{c} & y_{s}^{c} & z_{s}^{c} & \phi_{s}^{c} & \theta_{s}^{c} & \psi_{s}^{c} \end{array}\right]}^{T}$. The function *f* computes the re-projection error for each one of the markers; that is, the difference between the real observation and the projection of the marker according to the variable *x* and the calibration parameters of the camera. The problem is solved with a Levenberg-Marquardt [30] iterative algorithm available in the Ceres library [26]. As with all iterative methods, it needs an initial guess of the variables, which in our case is the predicted relative pose according to the EKF filter described previously.

In addition to the pose estimate, it is essential to have an estimate of the associated uncertainty, so that the pose information can be adequately used in a navigation filter. A first-order approximation of the pose covariance $\Sigma_{x^{*}}$ can be computed from the assumed covariance $\Sigma_{u}$ of the pixel location of the beacons in the image and the Jacobian $J\left(x^{*}\right)=\frac{\partial u}{\partial x}\left(x^{*}\right)$ that relates small changes in the pose parameter with small changes in the observations. The Levenberg–Marquardt implementation used computes and provides this Jacobian at the end of the minimization. The pose covariance estimate is given by:

(33) $$\Sigma_{x^{*}}={\left(J{\left(x^{*}\right)}^{T}\Sigma_{u}^{-1}J\left(x^{*}\right)\right)}^{-1}\;$$

**Figure 9:**
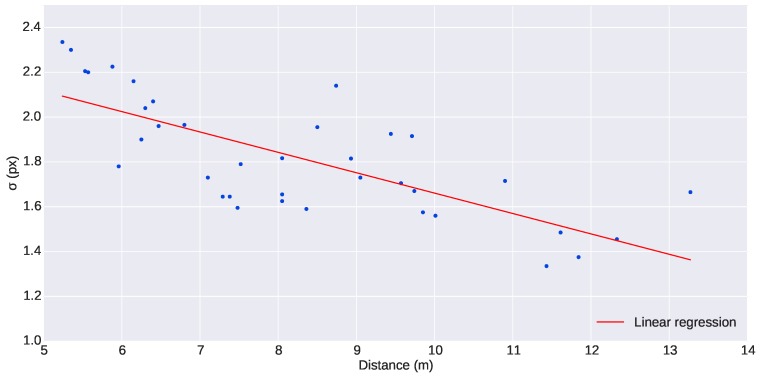
Evolution of the standard deviation of the Gaussian distributions fitted to the lights along distance.

The uncertainty in the localization of the lights in the image is inversely dependent on the distance of the beacon from the camera. The closer the beacon is to the camera, the larger the projected light disk will be in the image, thus leading to higher location uncertainty than far away beacons that appear in the image as small disks. In order to have an approximate value of this uncertainty, the size of the lights was analyzed from a set of selected images of the experiment presented in this paper. The beacons in the images were fitted to a 2D Gaussian distribution centered at *u*, with standard deviation *σ*, amplitude A and an offset $c_{0}$: $f(u,\sigma ,A,c_{0})$. The standard deviation of the 2D Gaussian distributions, *σ*, found can be used as an indicative value of the uncertainty of the location procedure. The experimental evidence from the mission presented in this paper (Figure 9) showed that the variation of *σ* is small enough to be considered constant within the range of distances of the experiments (5 to 12 m). A conservative mean value of σ=2 pixels was chosen for the standard deviation of both horizontal and vertical pixel uncertainties. The covariance used was:

(34) $$\Sigma_{u}=\left[\begin{array}{cc} \sigma^{2} & 0\\ 0 & \sigma^{2} \end{array}\right]=\left[\begin{array}{cc} 4 & 0\\ 0 & 4 \end{array}\right]\;$$

## 4. Results

**Figure 10:**
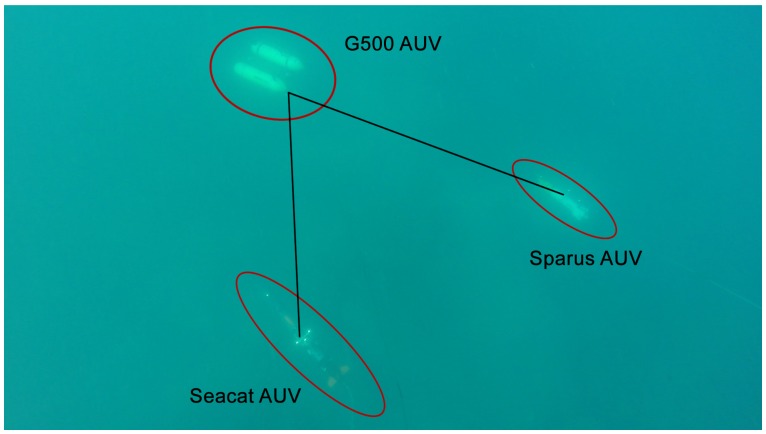
Image of G500, Sparus II and Seacat AUVs captured by a diver during a mission in Horta, Azores Islands, September 2015.

The performance of the light beacon tracking and pose estimation method was tested in several trials during the MORPH EU FP7-Project. The most relevant field testing took place in Sant Feliu de Guíxols, Spain, in March 2015 and in Horta, Azores Islands, in September 2015. This section presents the results obtained in one of the longest and most successful missions (Azores 15). The mission was performed by a total of five vehicles (four submerged and one at the surface) with the objective of mapping a vertical wall and the sea floor next to it. The Girona500 (G500) carried the omnidirectional camera and performed the role of the leading vehicle, while the Sparus II and Seacat AUVs [31] were the optical mapping vehicles, in charge of capturing high resolution imagery of the seabed (Figure 10).

Precise navigation data for all vehicles are fundamental for a good reconstruction of the multi-vehicle optical mosaic [32]. The formation control was performed with acoustic ranging following the MORPH guidelines [33,34]. The light beacons were used as an experimental technology being field tested at the time. As such, the tracking was not used in the formation control loop, but served as an important tool for online mission monitoring and to assist the data post-processing.

**Figure 11:**
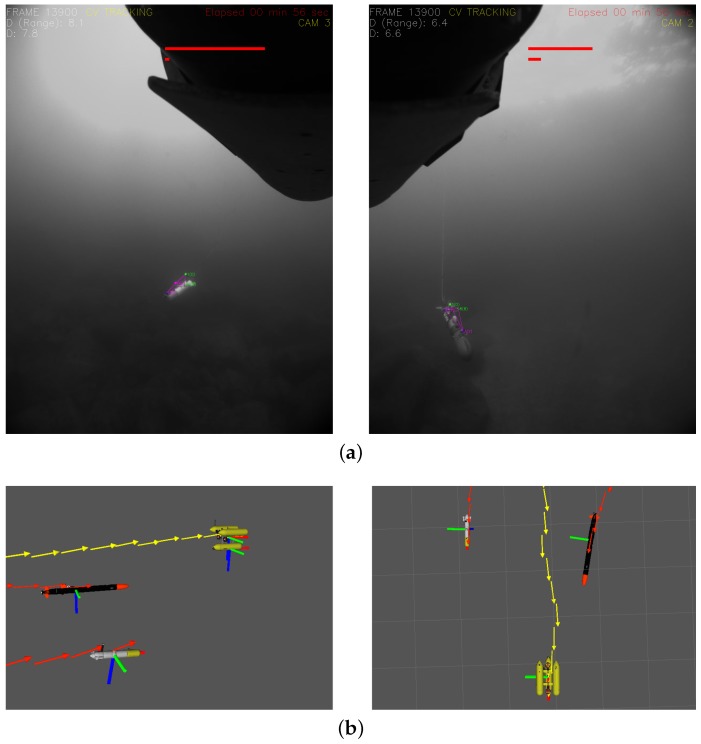
Snapshots of the system while tracking simultaneously two target vehicles. (**a**) Processed images from two independent cameras while the system is tracking two different target vehicles: Sparus (left) and Seacat (right); (**b**) left: lateral view of the reconstructed scene in the Rviz simulator; right: top view of the reconstructed scene in the Rviz simulator.

Figure 11 illustrates the optical tracking results. The system capabilities allow the reconstruction of the scene in a 3D viewer with a high update rate of both the position and orientation of the target vehicles, making it very easy for an operator to understand the development of the mission in real time or during replay.

**Figure 12:**
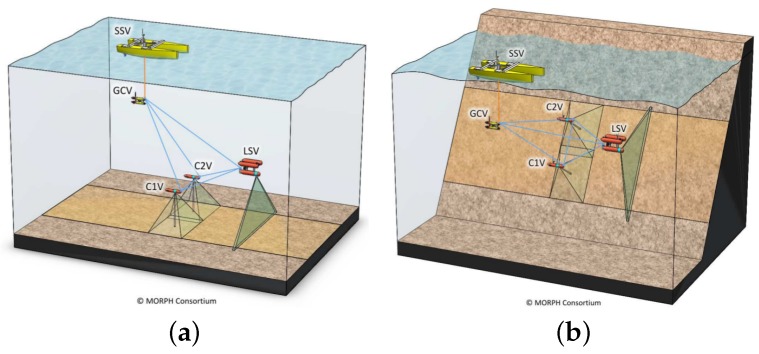
During the MORPH (Marine robotic system of self-organizing, logically linked physical nodes) project missions, the vehicles have the capability to change the formation geometry on-the-fly, adapting it to the changes in the terrain. When the formation navigates towards a wall, the two target vehicles, named in the figures C1V and C2V, change their altitude, which can result in one, or both, target vehicle(s) being out of the field of view of the omnidirectional camera and, therefore, without tracking capabilities. Source: MORPH Consortium. (**a**) Survey of a flat region. The two target vehicles navigate close to the bottom, below the leading vehicle; (**b**) Survey of a vertical wall. The two target vehicles have different altitudes, one or both of them above the leading vehicle.

The intended positions of the target vehicles with respect to the leading vehicle varied considerably during the mission. During most of the survey, the target vehicles were surveying the horizontal floor, and the desired positions were 5 m behind the G500 AUV and 5 m to both the left and right sides. However, when surveying the vertical wall, the two vehicles were commanded to move to the same side of the G500 and explore the wall with different altitudes (Figure 12). For this reason, along with the normal oscillation of the relative positioning due to the control system, the distance between the G500 and the target vehicles was not constant and varied between 4 and 18 m. The performance of the optical tracking system depends strongly on the distance between the camera and the target vehicles, the geometry of the relative positions of the projections of the light beacons on the image [19] and on the visibility conditions in the water, principally the turbidity and the presence of strong veiling light. The complete characterization of the environmental conditions under which the system can operate is undergoing assessment and is outside the scope of this paper. However, the tests reported in this paper were done in realistic conditions of visibility at sea. Although not measured precisely, the visibility was estimated by local divers to be in the 20–25 m range.

**Figure 13:**
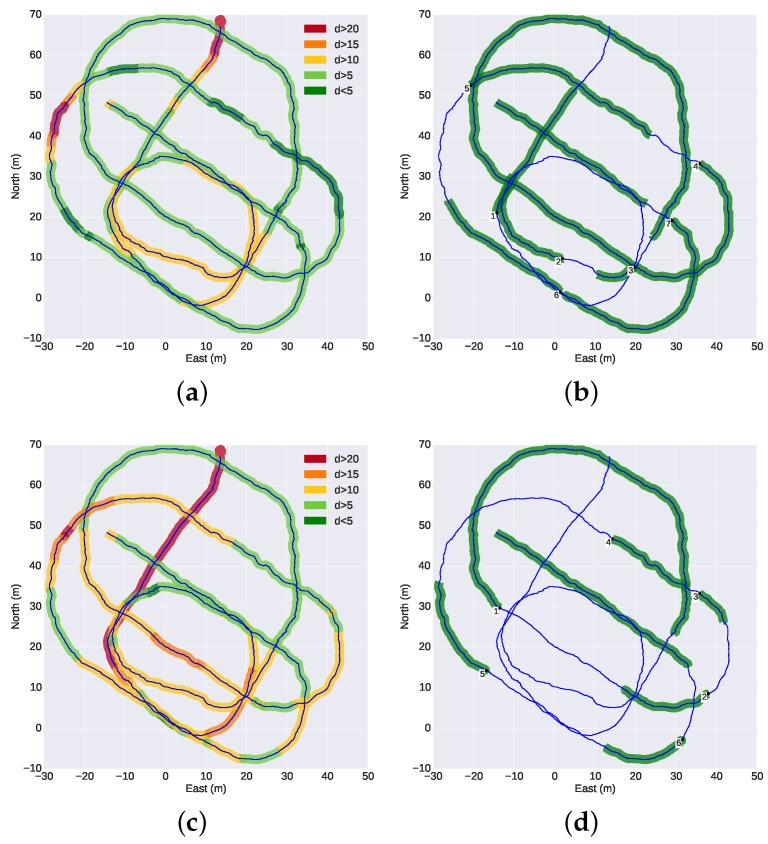
Top view of the trajectory of the G500 during the mission. The distances between G500 and target vehicles are color-coded (**a**,**c**); and the intervals of sustained tracking operation are marked in green (**b**,**d**). The points where the tracking was lost are numbered (**b**,**d**) for further analysis in Table 1 and Table 2. The survey of the vertical wall corresponds to the lower-left corner of the images. (**a**) Distance between G500 and Seacat; (**b**) intervals of optical tracking of the Seacat; (**c**) distance between G500 and Sparus; (**d**) intervals of the optical tracking of the Sparus.

**Figure 14:**
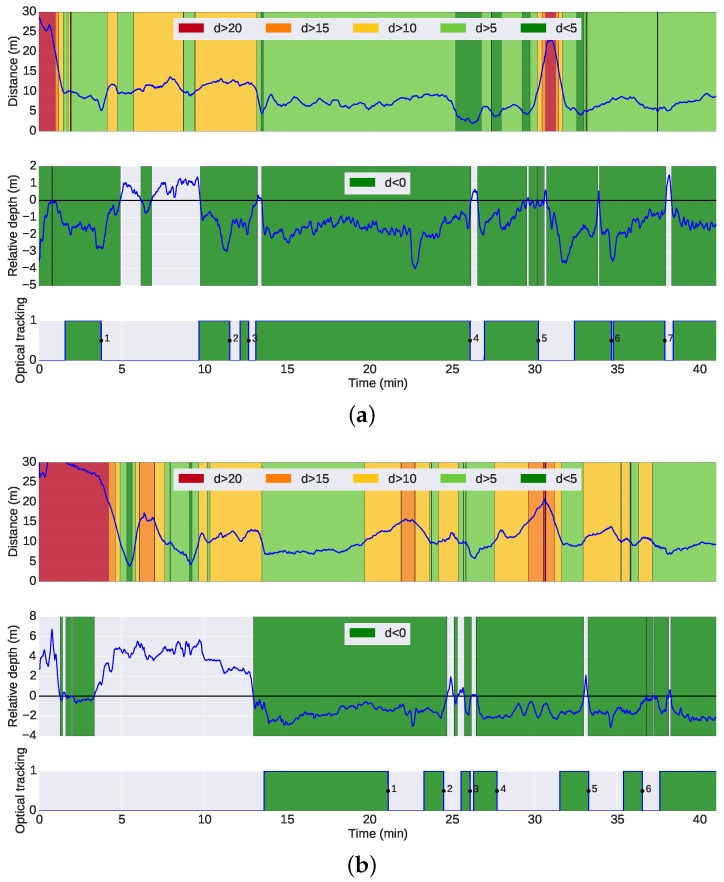
Analysis of intervals with optical tracking *versus* distance and relative depth between G500 and the target vehicles. Looking at the three plots in conjunction, it is easy to infer the necessary conditions for the operation of the optical tracking. (**a**) Top: analysis of the distance between G500 and Seacat over time; middle: analysis of the relative depth between G500 and Seacat over time. Negative values mean that Seacat was below G500; thus, the optical tracking was plausible. Bottom: intervals of optical tracking. The points where the tracking was lost are numbered for further analysis in Table 1; (**b**) Top: analysis of the distance between G500 and Sparus over time; middle: analysis of the relative depth between G500 and Sparus over time. Negative values mean that Sparus was below G500; thus, the optical tracking was plausible. Bottom: intervals of optical tracking. The points where the tracking was lost are numbered for further analysis in Table 2.

Figure 13 presents a top or planar view of the trajectory of the G500 during the mission. The figures on the left are color-coded with the distances between the G500 and the target vehicles, while the figures on the right show the intervals of the sustained operation of the optical tracking. The trajectories and inter-vehicle distances were computed from offline optimization using all navigation data available [32]. During some parts of the mission, especially during the wall survey, the target vehicles were flying above the leading vehicle and, thus, outside the field of view of the camera. On such occasions, it was not possible to perform the tracking, even when the relative distance between the vehicles was small. Figure 14 illustrates this issue: the top plot shows the distance between the G500 and the target vehicles similarly to Figure 13; the middle plot presents the relative depth between the vehicles; the bottom plot highlights the intervals of sustained operation of the optical tracking. An initial insight into the performance of the optical tracking can be inferred from Figure 14. The plots show that under the conditions of the experiment, the tracking system performs adequately when the target vehicles navigate below the camera vehicle at distances less than 10 m, with intermittent operation for distances between 10 and 15 m. For distances of more than 15 m, the operation is unreliable. The mission was performed over a 40-min period at noon, under cycling illumination conditions of overcast cloud and direct strong sunlight. The G500 AUV was navigating at a depth between 7 and 10 m, where the Sun still causes reflections on the body of the vehicles and decreases the visibility of the light beacons. Although not tested, it is safe to assume that performance would improve even further at deeper depths or night-time conditions.

**Figure 15:**
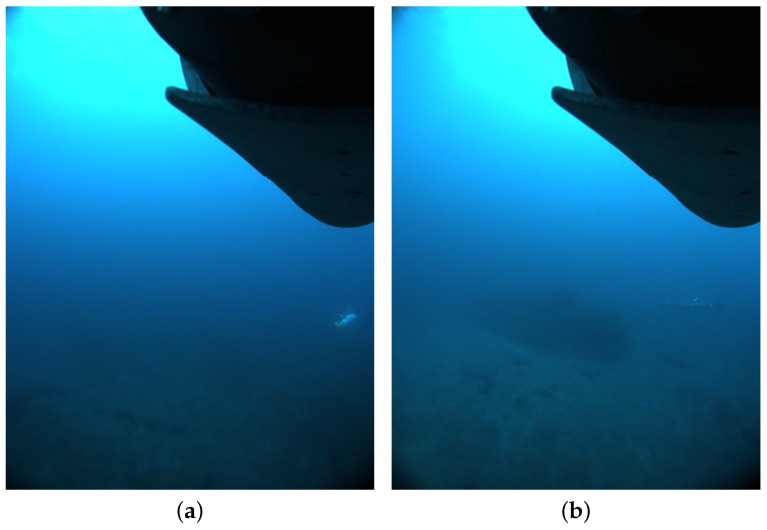
Two instances where the tracking system lost the target vehicles due to the visibility conditions. The lights at the distance of the vehicles were too weak for the system to discern them and keep the tracking running. (**a**) Sparus at approximately a 13.2-m distance; (**b**) Seacat at approximately a 12.4-m distance.

Regarding the failure modes, Figure 15 shows two examples of the limit conditions of operation. Both images correspond to loss of tracking after a long interval of operation. In both cases, the distance between the camera and the vehicles was a decisive factor. The limit distance is determined not only by the visibility conditions, but also by the resolution of the camera, which affects the apparent size of the light disk. In these images, the disks are less than two pixels in diameter. This was further compounded with the effects of ambient light and, in the case of Seacat, with the poor geometry of the image projected light beacons, where two beacon disks are overlapping.

Table 1 and Table 2 show the failure modes for each instant where tracking was lost during the mission. Three main reasons appear: (1) *distance*, the distance between the vehicles was too big and tracking became unreliable; (2) *FOV*, the target vehicle navigated higher than the camera-vehicle, and thus, it moved outside the camera field of view, or occlusions appeared between the markers and the body of the target vehicle; (3) *geometry*, the combination of the pose of the target vehicle with the geometry of the markers made it very difficult to estimate the pose correctly.

Let us now consider one of the longest sections with tracking capabilities for both vehicles. In Table 3, we compare the information of the relative navigation between the leading vehicle and the target vehicles in three different cases: (1) using only acoustic ranges; (2) using USBL updates; or (3) using the light beacon tracking system. One of the most important limitations of underwater communications using acoustics is that for a reliable and stable communication, only one emitter is allowed to send messages at any given time. For this reason, when several vehicles co-exist, their access to the media has to be scheduled using a time division media access (TDMA) protocol, assigning to each vehicle a safe slot of time for sending messages. In the case of the mission analyzed, due to the number of vehicles involved and all of the different uses of the acoustic channel, each one of the vehicles was able to send messages every 5 s. We can observe from Table 3 that the information provided by the light beacon system is the most complete, as it reports data on both the position and orientation of the target vehicle, unlike USBL or range-only systems, which report only relative position or distance. The number of updates received is another significant difference: acoustic-based systems only provided 122 updates during the period studied, while the light-beacon system reported up to 90-times more updates. This is due to the constraints on the acoustic or range-only communications when multiple vehicles are in the water, while the optical system is independent of the number of vehicles in the water.

**Figure 16:**
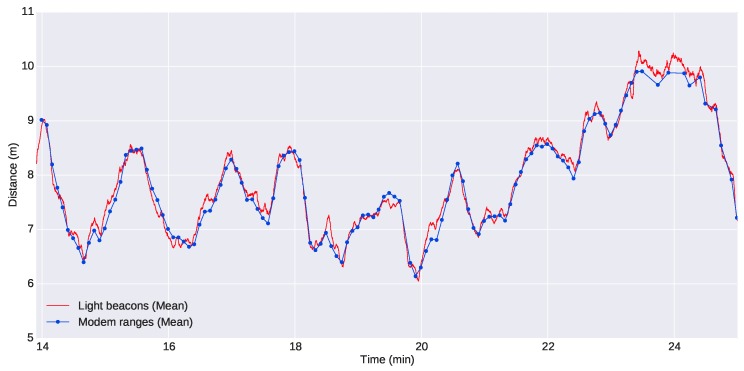
Comparison of the distance between the G500 and the Seacat estimated using light beacons and acoustic ranges.

Figure 16 shows a comparison of the distance between the G500 and the Seacat AUV computed through acoustic ranges and using the light beacon system: the two online localization systems that were available for the leading vehicle. Looking at the figure, it is clear that there is a high level of agreement between the two systems, and there is no apparent offset between the different approaches. We can also see the difference in the number of updates.

**Figure 17:**
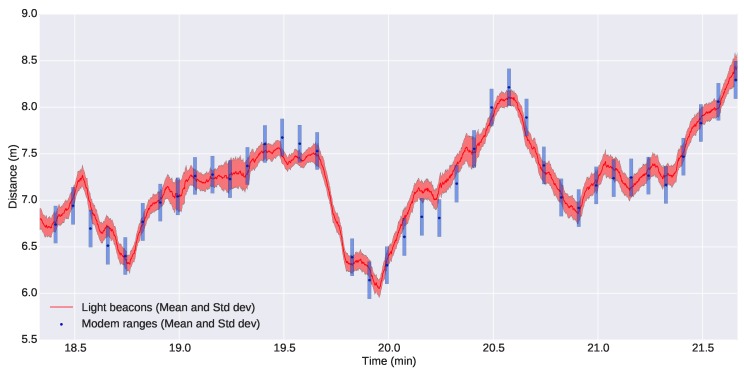
Detailed comparison between the distance estimated using light beacons and acoustic ranges along with their uncertainties.

Figure 17 shows a zoomed section of the former plot, but including the uncertainties of the measurements. The uncertainties of the poses computed through the optical system have been calculated according to Section 3, while the uncertainty of the acoustic ranges has been estimated as a fixed value obtained from computing the standard deviation between data from the acoustic ranges and the estimated distances using the optical system. For both cases, we have chosen conservative values: a standard deviation of 0.2 m for the acoustic ranges and a standard deviation of two pixels for localization of the lights in the images, as explained in Section 3.2.2. We can observe from the figure that the optical system is very competitive against acoustic ranges and even for the worst cases does not exceed the 0.2-m standard deviation of the acoustic ranges.

**Figure 18:**
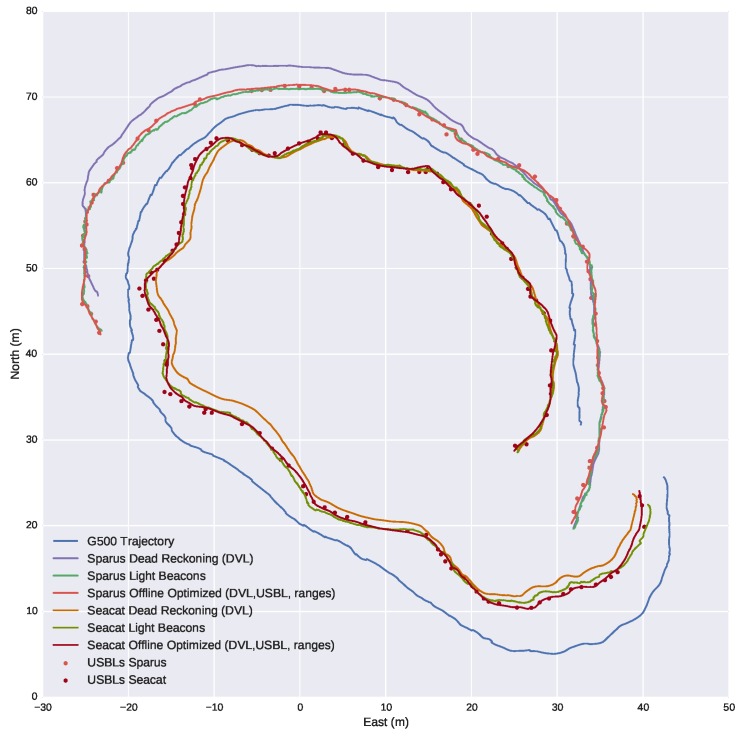
Comparison between the estimated position of target vehicles using light beacons and the position according to their navigation systems before and after its optimization.

The last two plots provide evidence of the accurate behavior of the tracking system. However, they are only a comparison in one dimension, whereas the system provides full position information. With the aim of achieving a more complete comparison, Figure 18 shows the navigation of the three vehicles according to different navigation systems. Different conclusions can be drawn from the figure:

- Comparing the trajectories computed by the internal navigation systems of each vehicle, labeled as *dead reckoning*, with the USBL updates and the trajectories computed by the other systems, we can infer that the internal navigation systems have a significant drift over time, especially Sparus, due to the fact that they are based on inertial sensors and DVL.
- The distances between the USBL updates and the trajectory computed by the light beacon system are small. This fact makes clear that the computed trajectory is consistent with the USBL updates. It also becomes clear that the number of updates is significantly higher for the light beacon system, as seen previously.
- The navigation trajectories computed by the optical method and the offline optimization method have a high degree of agreement. From this fact, we can conclude that the light beacon system is consistent with the offline optimized trajectory of the vehicles. This trajectory was computed once the mission was finished, gathering the navigation data from the vehicles participating in the mission and the acoustic ranges and USBLs received, and is assumed to be the best navigation estimate we can compute without the light beacon system. The trajectory was computed with the algorithm described in [32].

**Figure 19:**
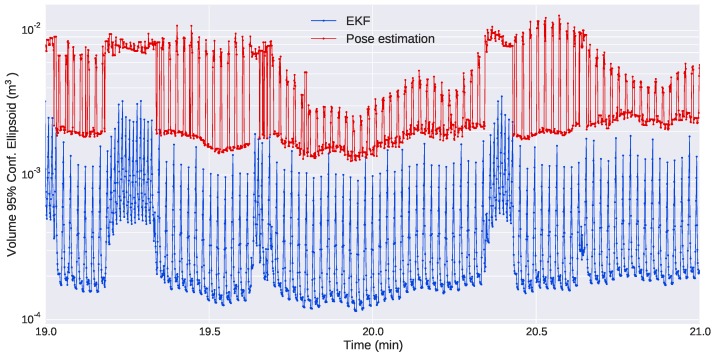
Volume of the ellipsoid that contains the position of the target vehicle with a probability of 95%. In red, the volume of the ellipsoid computed directly from the uncertainty given by the pose estimation module. In blue, the volume of the ellipsoid according to the uncertainty estimated by the EKF. The peaks in the red plot correspond to the pose estimates computed from only three markers, which have a greater uncertainty than the estimates computed from four markers. The peaks in the blue plot show how the uncertainty of the position grows until a new observation of the target pose is received.

Finally, Figure 19 show the evolution of the uncertainties in the position of the Seacat during a section of the mission. The plot shows the difference in the uncertainties of the estimates computed from three or four markers and how the uncertainty grows when the pose of the target vehicle only allows the sight of three of them. It is also evident how the use of the EKF allows a drastic reduction in the uncertainty of the estimates and, thus, improves significantly the performance of the optical system.

## 5. Conclusions

This paper has presented a complete method for the tracking of AUVs when navigating in close-range based on vision and the use of active light markers. While traditional acoustic localization can be very effective from medium to long distances, it is not so advantageous at short distances, when for safety reasons, it is necessary to achieve a higher precision and faster update rate. The use of the proposed system in such conditions allows the pose estimation of a target vehicle with high accuracy and speed. To provide the system with the best possible performance, the camera used in the experiments was an omnidirectional model, which provides a coverage of 360${}^{\circ }$ in the horizontal plane and allows the system to track vehicles in different positions simultaneously.

The system was tested in mapping missions in real sea conditions. The results presented focused on a mission in which three vehicles were involved: a leader vehicle at the front of the formation and two AUVs at the back for capturing images of the seafloor. These last two were fitted with one set of light beacons each, while the leader was equipped with the omnidirectional camera. The control of the formation was performed using acoustic ranging, but the light beacons enabled the possibility of reconstructing their navigation and comparing them to their own navigation and acoustic systems. As expected, the results showed that the system performs adequately for vehicle separations smaller than 10 m, while the tracking becomes intermittent for longer distances due to the challenging visibility conditions underwater.

The navigation reconstructed from the output of the light tracking system shows a high level of agreement with the navigation computed from the vehicles’ navigation systems and acoustic ranging. In addition, when compared to a multi-vehicle setup with strong constraints on the use of the acoustic channel, the light beacon system is able to provide an enormous increase in the frequency of the updates (90-fold in the case of our test setup). It can also provide information about the orientation of the target vehicles, which most common acoustic systems cannot achieve without using the explicit data communication of sensor readings.

As a central conclusion, the use of light beacon technology for the pose estimation of underwater vehicles can be considered to be at a technology readiness level of at least six, since this paper presents a working prototype operating in a relevant/operational environment. This work also contributes by raising the feasibility of active light markers for complex cooperative underwater operations in close range, such as mapping missions for 3D environments or cooperative intervention missions.

## Figures and Tables

**Table 1:** Instants with a loss of visual tracking for Seacat and its failure mode. Points are numbered according to Figure 13b and Figure 14a.

Point	1	2	3	4	5	6	7
Failure mode	Geometry	Distance	Distance	FOV	Distance	Abrupt change in depth	FOV

**Table 2:** Instants with loss of visual tracking for Sparus and its failure mode. Points are numbered according to Figure 13d and Figure 14b.

Point	1	2	3	4	5	6
Failure mode	Distance	Distance + Geometry	Occlusion with Seacat	Distance	Distance	Distance

**Table 3:** Updates of relative navigation position received by Girona500 about Seacat during an interval of a duration of 11 min 21 s. USBL, ultra-short baseline system.

Localization system	Acoustic Ranges	USBLs	Light Beacons
Updates	122	122	10894
Information	Distance	Position	Position and orientation

## Supplementary Materials

The following are available online at http://www.mdpi.com/1424-8220/16/4/429/s1.
